# Mouth breathing reduces oral function in adolescence

**DOI:** 10.1038/s41598-024-54328-x

**Published:** 2024-02-15

**Authors:** Yukako Masutomi, Takaharu Goto, Tetsuo Ichikawa

**Affiliations:** Department of Prosthodontics and Oral Rehabilitation, Tokushima University Graduate School of Biomedical Sciences, 3-18-15 Kuramoto, Tokushima, 770-8504 Japan

**Keywords:** Health care, Medical research

## Abstract

Although humans breathe naturally through the nostrils, mouth breathing in children has recently gathered attention. In this study, we hypothesized that tongue function and its related maxillofacial morphology would affect breathing in adolescence. To verify this hypothesis, we examined the association between breathing patterns, including mouth and nasal breathing; oral functions, including tongue motor function; and craniofacial morphology during adolescence, which has not been investigated till date. C3-H, which indicates the anteroposterior position of the hyoid bone in relation to the third cervical vertebra, was significantly smaller in mouth-breathers than in nasal-breathers. Lip-closing force, tongue pressure, and masticatory efficiency were lower in the order of nasal-breathers, oronasal-breathers, and mouth-breathers, and the values for mouth-breathers were significantly lower than those for nasal-breathers. Tongue pressure alone was identified as a significant independent variable, with an odds ratio of 1.063 (95% confidence interval, 1.006–1.123; *p* < 0.05). Our results indicate a relationship between mouth breathing and the lip-closing force, tongue pressure, and masticatory efficiency, as well as the significance of tongue pressure on mouth breathing in adolescents. The findings highlight the importance of clarifying the pathophysiology of mouth breathing and its underlying causes.

## Introduction

Breathing is an important life function and is a crucial physiological parameter for assessing the health status of individuals^1–3^. Although humans breathe naturally through the nostrils^4^, mouth breathing in children has recently gathered attention. Children with mouth breathing possess poorer reading comprehension and working memory than those without mouth breathing^5^. Moreover, mouth breathing is associated with obstructive sleep apnea, which adversely affects growth and academic performance^6^. Therefore, addressing this issue has become increasingly important. From a dental perspective, mouth breathing is not only associated with oral dryness, dental caries, and periodontal disease but also with maxillofacial growth and malocclusion^7–9^. In children with a high incidence of mouth breathing, collaboration among pediatrics, otolaryngology, pediatric dentistry, and other related departments, with active intervention, is essential.

Mouth breathing is classified into two categories according to the presence of nasal obstruction, and mouth breathing without nasal obstruction is often referred to as habitual mouth breathing^10,11^. Multiple etiologies are involved in mouth breathing with nasal obstruction, including allergic rhinitis, adenoid and tonsil hypertrophy, and nasal septal deviation^12,13^. However, the etiology and mechanisms of habitual mouth breathing have been described less frequently and are often ambiguous in clinical practice. Nogami et al. reported that the percentage of children with suspected mouth breathing increased from < 20% at the age of 3 years to 40% at the age of 12 years, and the percentage increased with age^14^. This is thought to be because the incidence of allergic rhinitis increases with age and tonsil hypertrophy peaks at 6–7 years of age. However, an increasing proportion of children with mouth breathing have mild or no nasal obstruction and are diagnosed with habitual mouth breathing^15^.

Delayed development and inadequate acquisition of oral functions such as mastication, swallowing, and articulation, have been recently recognized as issues^16^. In Japan, the importance of oral health management from early childhood has been advocated^17,18^, and a new clinical concept titled "developmental insufficiency of oral function" has been proposed, which has been recognized by the Japanese insurance system since 2018^19^. In recent years, the association between oral function and mouth breathing in children has been increasingly researched; however, most studies on the subject have examined the relationship with lip-closing force. Saitoh et al. reported that the lip-closing force increased in children aged 3–6 years and reached a plateau at 7–12 years of age^20^. Nogami et al. reported that the prevalence of lip incompetence in children aged 3–12 years was approximately 30%, and the condition was related to the orofacial morphology and mouth breathing^14^. In a study of children aged 6–12 years by Basheer et al., all children with mouth breathing exhibited incompetent lips^21^. Although a correlation between the lip-closing force or incompetent lips and mouth breathing has been reported, no mechanism has been proposed to justify incompetent lips as a direct cause of mouth breathing. In addition to the lip-closing force, tongue motor function plays an important role in sucking in infancy and mastication in later life. Mastication, in which various oral organs, including the lips and tongue, cooperate with each other, is considered closely related to mouth breathing. However, this association has not yet been fully investigated. Regarding the correlation between mouth breathing and masticatory efficiency, Sugawara et al.^22^ and Sakaguchi^23^ reported a significant prolongation of chewing time and a decrease in masticatory efficiency during acute nasal obstruction. However, these studies compared the effects of artificial nasal obstruction using nose clips with those of normal conditions in adults. Thus, little information is available on children with habitual mouth breathing.

In order to figure out what triggers mouth breathing as the ultimate goal of research project, we hypothesized that the factors that cause delayed development and inadequate acquisition of oral functions, especially tongue movement functions, may be related to factors that lead to mouth breathing. We believed that during the growth process from infancy to early childhood, various environmental factors such as food and childcare prevent sufficient activity of the tongue and surrounding muscles during sucking and mastication. Mouth breathing subsequently develops due to the lack of coordination between the tongue, lip, and mandibular movements. Its continuation leads to the establishment of mouth-breathing through the skeletal development of the lower face, which is disadvantageous to nasal breathing, during school-age and adolescence. This hypothesis is based on Moss's functional-matrix theory^24^, which states that the functional development of muscles and soft tissues greatly influences bone formation. As mentioned above, tongue function plays an important role from sucking in infancy to chewing and swallowing later in childhood. However, oral functions including tongue function, maxillofacial morphology, and their relationship in mouth-breathers have not been clarified.

In this study, we hypothesized that tongue function and its related maxillofacial morphology would affect breathing in adolescence to figure out the cause of oral breathing as the ultimate goal of our research project. To verify this hypothesis, we examined the association between breathing patterns, including mouth and nasal breathing; oral functions, including tongue motor function; and craniofacial morphology during adolescence, which has not been investigated till date.

## Results

Table 1 presents the percentages of each breathing pattern among the 103 participants. Twenty-one participants (20.4%) were mouth-breathers, 25 participants (24.3%) were oronasal-breathers, and 57 participants (55.3%) were nasal-breathers. The male-to-female ratios in all groups were similar.

**Table 1:** Percentage of patients presenting each breathing-pattern.

	Male	Female	Total
Mouth-breathers	9 (8.7%)	12 (11.7%)	21 (20.4%)
Oronasal-breathers	14 (13.6%)	11 (10.7%)	25 (24.3%)
Nasal-breathers	26 (25.2%)	31 (30.1%)	57 (55.3%)

Table 2 shows the incidence of malocclusions in each breathing group. Overall, reverse bite, open bite, maxillary protrusion, overbite, crossbite, and crowding were observed in 1, 5, 8, 8, 18, and 17 patients, respectively. Among the nasal-breathers, 30 (29.1%) had a normal bite and 27 (26.2%) had malocclusion. In the mouth- and oronasal-breather groups, 7 (6.8%) and 9 (8.7%) patients had a normal bite and 14 (13.6%) and 16 (15.6%) had malocclusions, respectively. However, the chi-square test showed no significant association between malocclusion and breathing patterns.

**Table 2:** Prevalence of malocclusion in each breathing-pattern group.

	Normal occlusion (n = 46)	Malocclusion (n = 57)
Mouth-breathers (n = 21)	7 (6.8%)	14 (13.6%)
Oronasal-breathers (n = 25)	9 (8.7%)	16 (15.6%)
Nasal-breathers (n = 57)	30 (29.1%)	27 (26.2%)

Table 3 presents the craniofacial morphometry results. The SNA and SNB angles were smaller in mouth-breathers than in nasal-breathers; that is, the upper and lower jaws tended to be retracted. However, the differences were not statistically significant. ANB angle was not also statistically significant in each breathing group. The FMA and ANS-Me values were larger in the order of nasal-breathers, oronasal-breathers, and mouth-breathers, which were the so-called “high-angle cases”. These values were significantly higher in mouth-breathers than in nasal-breathers. MP-H, which indicates the vertical position of the hyoid bone relative to the mandible, was larger in mouth-breathers than in nasal-breathers. This suggests a lower position of the hyoid bone relative to the mandible; however, the difference was not statistically significant. C3-H, which indicates the anteroposterior position of the hyoid bone in relation to the third cervical vertebra, was significantly smaller in mouth-breathers than in nasal-breathers. This suggests that the hyoid bone in mouth-breathers was significantly closer to the cervical vertebra and posterior to the cranium compared to that in nasal-breathers. PNS-P, which indicates the length of the soft palate, was significantly smaller in mouth-breathers than in nasal-breathers. In consideration of the sex differences, the results were compared by standardizing with a mean value of 0 and standard deviation of 1. FMA, ANS-Me, C3-H, and PNS-P were significantly different between the mouth-breathing and nasal-breathing groups (Supplement File S1).

**Table 3:** Cephalometric parametric values in each breathing-pattern group.

	Mouth-breathers	Oronasal-breathers	Nasal-breathers	*P*-value
SNA	78.6 ± 3.7	79.5 ± 3.7	79.7 ± 3.8	0.229
SNB	77.4 ± 4.1	77.5 ± 3.3	78.4 ± 3.6	0.511
ANB	1.2 ± 2.1	1.9 ± 2.1	1.3 ± 2.4	0.812
FMA	29.3 ± 5.7	27.3 ± 5.8	25.2 ± 5.9	0.014*
ANS-Me	72.8 ± 5.1	72.4 ± 5.7	69.8 ± 4.9	0.021*
MP-H	13.1 ± 4.8	12.7 ± 5.2	12.0 ± 5.0	0.387
C3-H	35.6 ± 3.9	37.0 ± 4.4	37.9 ± 4.5	0.023*
Me-H	41.5 ± 5.4	40.8 ± 6.3	41.5 ± 5.6	0.821
PNS-P	32.9 ± 5.5	34.1 ± 6.0	36.2 ± 5.2	0.021*

*Comparison between mouth and nasal-breathers. Mann–Whitney U-test, *p* < 0.05.

Table 4 shows the mean and standard deviation of oral-function values for each breathing group. For all measurements, the values for mouth-breathers were lower than those for nasal-breathers. Particularly, lip-closing force, tongue pressure, and masticatory efficiency were lower in the order of nasal-breathers, oronasal-breathers, and mouth-breathers, and the values for mouth-breathers were significantly lower than those for nasal-breathers.

**Table 4:** Oral-function values in each breathing-pattern group.

	Mouth-breathers	Oronasal-breathers	Nasal-breathers	*P*-value
Maximum occlusal force	872.2 ± 380.4	1015.0 ± 502.7	967.3 ± 442.9	0.458
Occlusal contact area	24.8 ± 9.9	31.0 ± 14.8	28.8 ± 12.2	0.206
Lip-closing force	7.3 ± 2.5	7.6 ± 2.4	8.7 ± 2.6	0.020*
Tongue pressure	28.9 ± 8.9	30.5 ± 8.4	34.4 ± 9.4	0.016*
Masticatory efficiency	141.4 ± 45.6	172.3 ± 59.3	182.8 ± 65.3	0.008*

*Comparison between mouth and nasal-breathers. Mann–Whitney U-test, *p* < 0.05.

Table 5 presents the results of the binomial logistic regression analysis after adjusting for age. The variance inflation factors for the maximum occlusal force and occlusal contact area were 9.96 and 10.17, respectively, and the maximum occlusal force was selected because it was a functional parameter. Tongue pressure alone was identified as a significant independent variable, with an odds ratio of 1.063 (95% confidence interval, 1.006–1.123; *p* < 0.05). The goodness of fit of the model was confirmed using the χ2 and Hosmer–Lemeshow tests, and percentage of correct classification (Model χ2 test, *p* < 0.05; Hosmer–Lemeshow test, 0.067; percentage of correct classification, 66.7%).

**Table 5:** Results of the binomial logistic regression analysis after adjusting for age.

	B	SE	Wald	Odds ratio	95% CI		*P*- value
					Lower	Upper	
Tongue pressure	0.061	0.028	4.662	1.063	1.006	1.123	0.031*

*P < 0.05; SE, standard error. CI, confidence interval. Model x^2^ test *P* < 0.05; Hosmer–Lemeshow test = 0.067; Percentage of correct classification = 66.7%.

## Discussion

Of the 103 participants included in this study, 21 (20.4%) were mouth-breathers. The prevalence of mouth-breathers has been reported in several previous studies; Humphrey et al. studied 1033 infants aged 2–5 years, of whom 220 (21%) were mouth-breathers^25^. In a study of children aged 4–6 years, Kogue et al. reported a prevalence of 22.8%^26^, whereas Shindo reported a prevalence of 29.0% in a survey of elementary-school students^27^. Thus, most studies on the proportion of mouth-breathers have focused on young children, and studies on adolescents (age > 13 years) are lacking. As mouth breathing has been gathering attention not only in medicine and dentistry but also in various other fields, the present study’s identification of mouth breathing among adolescents is noteworthy. The prevalence of mouth-breathers in this study was similar to that reported in previous studies on infants and elementary school children, suggesting that mouth breathing may have appeared at an early age.

Regarding the association between mouth breathing and malocclusion, previous studies have reported a higher prevalence of malocclusion in mouth-breathers than in nasal-breathers^28,29^. Although this tendency was observed in our study, the association was not statistically significant. Genetic factors have been considered to markedly influence the morphology of the dentition and occlusion; however, functional factors, such as breathing and habits, have also been reported as contributors^30^. Previous studies have reported a significant association between maxillary protrusion and mouth breathing^31,32^.

Evaluation of the craniofacial morphology revealed that in mouth-breathers, the inclination of the mandibular plane to the Frankfort plane and lower-face height were greater and the mandible was rotated posteriorly compared to those in nasal-breathers. These results are consistent with those of previous studies^33–35^. Regarding MP-H, which is an index of the vertical position of the hyoid bone, no significant difference was observed between nasal and mouth-breathers. Previous studies have reported that the hyoid bone of children with mouth breathing is positioned higher in relation to the mandible and cervical vertebrae compared to that in children with nasal breathing^36–38^; however, the hyoid bone in mouth-breathers was lower than that in nasal-breathers when the head was extended^39,40^, therefore, a consensus on this is lacking. Juliano et al. reported that the hyoid bone descends with age and that MP-H was not significantly different between mouth- and nasal-breathing children, but a significant difference was identified in adults^41^. Considering that this study included adolescents aged 12–25 years, age may have influenced the results. Regarding C3-H, which indicates the anteroposterior positional relationship of the hyoid bone, in this study, the hyoid bone was located closer to the cervical vertebrae and more posterior to the cranium in mouth-breathers than in nasal-breathers. In a study targeting participants aged 6–12 years, Chung et al. reported that mouth-breathers presented smaller C3-H values than nasal-breathers^37^; other studies have also reported similar results^38,42^. In this study, PNS-P, which indicates the length of the soft palate, was significantly smaller in mouth- breathers than in nasal-breathers. Although several previous studies have reported a relationship between PNS-P and obstructive sleep apnea syndrome^43,44^, the differences between mouth and nasal-breathers have not yet been investigated. Obstructive sleep apnea syndrome is characterized by sleep-disordered breathing due to a large PNS-P. In this study, PNS-P was smaller in mouth-breathers, possibly because the soft palate extends with age, and the mean age of mouth-breathers in this study (15.6 ± 1.8 years) was lower than that of nasal-breathers (16.7 ± 3.0 years). A more detailed study of this aspect, including anatomical structures, is warranted.

Regarding oral function, the lip-closing force, tongue pressure, and masticatory efficiency were significantly lower in the mouth-breathing group than in the nasal-breathing group. Saitoh et al. reported that incompetent lips are representative of the physical appearance of mouth breathers^45^, with several similar reports showing a significant association between the two. A shift in the breathing pattern from nasal to mouth breathing causes buccal tension, lip laxity, tongue hypotonia, and decreased muscle activity in the posterior neck and anterior temporal muscles^46–48^. In this study, mouth-breathers presented a decrease in oral functions compared to nasal-breathers. Because the measurement of oral function is a self-administered test, the reliability of the measured values is low in younger patients. In this study, a decline in oral function was observed in mouth-breathers during adolescence, which is the earliest age when self-administered tests are possible.

The present study revealed that adolescent mouth-breathers have a posteriorly positioned hyoid bone, a posteriorly rotated mandible, and decreased oral functions, such as lip-closing force, tongue pressure, and masticatory efficiency, compared to nasal-breathers. The occurrence of these maxillofacial morphological and functional phenomena in adolescent mouth-breathers indicate that issues in early childhood may influence the condition, considering that the percentage of mouth-breathers in adolescence was not different from that in early childhood, and that no significant relationship was found between mouth breathing and malocclusion in adolescence. Furthermore, although several studies have reported an association between mouth breathing and lip-closing force, in this study, logistic regression analysis revealed a significant effect of tongue pressure on mouth breathing among adolescents. Haishima et al. reported the significance of tongue movements during infancy: infants are natural nasal-breathers who effectively perform sucking movements by fixing the hyoid bone in the anterosuperior position and suppressing the mouth-opening function of the function of the suprahyoid muscle group to open the mandible, thereby stabilizing the mandible and enabling rapid wave-like movements of the tongue^49^. Therefore, the tongue plays a vital role during early infancy. However, concern exists regarding the inadequate development of sucking movements or tongue muscles because of an inappropriate eating environment and childcare posture from infancy to early childhood. To clarify the triggers of mouth breathing as the ultimate goal of this research project, we hypothesized that the living environment may decrease the strength of the muscles surrounding the hyoid bone, causing dysfunction in the posterior positioning of this bone, disruption of the tongue’s contact with the soft palate, backward rotation of the mandible, mouth breathing, jaw closing muscle inhibition^50^, and reduction in the lip-closing force, all of which are interrelated. We believe that our results, including the results of the logistic regression analysis, support our research hypothesis focused on the correlation between mouth breathing and tongue function, which had not been examined till date. Although this was a cross-sectional study in adolescents, it was the first step to assess our hypothesis that tongue problems in infancy affect the acquisition of maxillofacial morphology and oral function in adolescence. Future cross-sectional studies with a wider age range, including infancy and early childhood, as well as long-term longitudinal studies are required. The present study included 21 mouth-breathers and 57 nasal-breathers, and post-hoc analysis was performed using a free software (G*power 3.1.9.7, Heinrich-Heine-University, Düsseldorf, Germany). The results showed that α = 0.05, the effect size derived from the mean and standard deviation was 0.73, and the power was 0.81, suggesting that the sample size of this study was acceptable. However, the effect of bias on the number of participants cannot be ruled out because the sampling in this study was based on participants attending a single dental clinic. Therefore, in future, the population size should be increased to eliminate this effect considering stratification by age. Breathing patterns were assessed using a questionnaire for comparison with previous data. Quantitative evaluation methods for mouth breathing include measuring the amount of nitric oxide secreted from the sinus mucosa and separately evaluating the air in nasal and mouth-breathers; however, both methods require the use of a mask, hindering the measurement in a more physiological and natural condition in children. Understanding and responding to breathing patterns has become increasingly important in various fields, including medicine and dentistry, and the development of a more objective and simpler quantitative evaluation method for mouth breathing is expected.

## Conclusion

In adolescence, compared to nasal-breathers, mouth-breathers have morphological differences (a more posteriorly positioned hyoid bone and posteriorly rotated mandible) and significantly lower oral functions (such as lip-closing force, tongue pressure, and masticatory efficiency), with tongue pressure significantly influencing mouth breathing. Our results indicate a relationship between mouth breathing and the lip-closing force, tongue pressure, and masticatory efficiency, as well as the significance of tongue pressure on mouth breathing in adolescents. The findings highlight the importance of clarifying the pathophysiology of mouth breathing and its underlying causes.

## Methods

### Participants

A total of 103 patients (49 males and 54 females; age, 12–25 years [mean, 16.6 ± 2.7 years]) who underwent regular checkups at a general dental clinic since childhood and visited the clinic between March 2021 and April 2022 after the onset of second molar eruption were included in this study. Patients undergoing orthodontic treatment and those who have completed orthodontic treatment were also excluded. This study was approved by the Ethics Review Committee of the Tokushima University Hospital for Life Science and Medical Research (Approval No., 3912) and was conducted in accordance with the Declaration of Helsinki. Informed consent was obtained from all participants or their guardians.

### Assessments

Mothers and children completed an interview sheet regarding basic attributes such as sex, age, and lifestyle habits. Additionally, breathing patterns, malocclusion, oral function, and craniofacial morphology were assessed as follows.

### Breathing pattern

Breathing patterns were assessed using a questionnaire consisting of two questions based on the report by Kogue et al.^26^, as follows: (1) Do you often open your mouth during the day, and (2) Do you sometimes sleep with your mouth open? Those who answered "yes" to both questions were classified as “mouth-breathers,” those who answered "no" to both questions were classified as “nasal-breathers,” and the remaining were classified as “oronasal-breathers.” We first interviewed the examinees themselves, and if they answered "I don't know," we interviewed their parents or guardians to obtain their opinions.

### Malocclusion

The presence of reversed occlusion, open bite, maxillary protrusion, overbite, crossbite, and crowding was assessed by analyzing plaster casts according to the Japanese Association of School Dentists' criteria^51^. All the assessments were performed by a single pedodontist (Y.M.).

### Oral function

The maximum occlusal force, occlusal-contact area, lip-closing force, tongue pressure, and masticatory efficiency were assessed to evaluate oral function. The maximum occlusal force and occlusal-contact area were measured using Dental Prescale II (GC, Tokyo, Japan) with the participants seated such that the head was not fixed to the headrest, and the Frankfurt plane was parallel to the floor. Participants were instructed to bite the Prescale sheet for 3 s, and the maximum occlusal force was measured using a bite-force analyzer (GC, Tokyo, Japan).

Lip-closing force was measured using Lippule‐kun® (Shofu, Kyoto, Japan). An oral screen-like appliance connected to the device was placed in the oral vestibule. During the measurements, participants sat with the Frankfort plane parallel to the floor. They were instructed beforehand regarding the following measurement conditions: resisting traction using the muscle strength of the lips alone, not resisting traction using a sucking force, and not bending the head and body forward while resisting. The maximum force applied to pull the oral screen-like appliance horizontally was measured as the lip-closing force.

Tongue pressure was measured using a tongue pressure-measuring instrument (JMS Co., Hiroshima, Japan). A disposable balloon probe was inserted into the participants’ mouth, and participants were instructed to hold the probe gently with their front teeth and press their tongue against the pressure-sensing portion of the probe with maximum force for 7 s. The maximum pressure on the balloon was recorded as the tongue pressure, and the mean of three measurements was used as the representative value.

Masticatory efficiency was assessed using a masticatory ability testing system (Gluco Sensor GS-II; GC Co., Tokyo, Japan). The participants were asked to chew 2 g of gummy jelly for 20 s on their habitual chewing side and then gently expectorate it with 10 mL of water. The glucose level in the eluted filtrate, which was measured using the abovementioned device, reflected the masticatory efficiency.

### Craniofacial morphology

**Figure 1:**
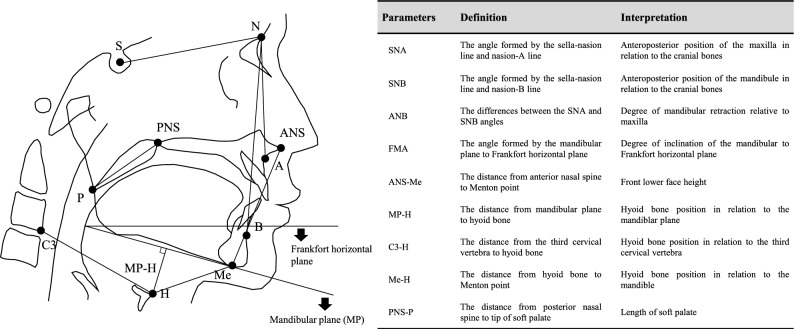
Cephalometric parameters.

The craniofacial morphology was assessed using a lateral cephalogram (Veraviewepocs 3DF X550 100CP; Morita, Osaka, Japan). Participants were instructed to stand with the Frankfurt plane parallel to the floor. Ear rods were inserted into the bilateral external auditory canals, and the head was fixed. The radiograph was obtained after the participants were asked to swallow and bite in the intercuspal position. A dentist (T.G.) trained in cephalometric analysis plotted the measurement points, and another skilled dentist (Y.M.) confirmed the results. Prescribed distances and angles were measured using an analysis software (Win Ceph Ver. 11; Rise, Miyagi, Japan), as shown in Fig. 1. In previous reports based on cephalometric measurements, airflow obstruction by the adenoids was suspected in patients with nasopharyngeal-airway diameter < 5 mm, and airflow obstruction due to palatine-tonsil hypertrophy was suspected in patients with mid-pharyngeal airway diameter ≥ 15 mm^52,53^. Therefore, we excluded patients who met these criteria to eliminate participants with mouth-breathers due to mechanical airflow obstruction and select habitual mouth-breathers.

### Statistical analysis

A chi-square test was used to examine the relationship between the presence of malocclusion and breathing pattern, and the Mann–Whitney U test was used to compare the mouth-breathing and nasal-breathing groups. Using two breathing patterns, mouth breathing and nasal breathing as dependent variables (0: mouth breathing, 1: nasal breathing), and four oral functions-maximum occlusal force, occlusal contact area, lip-closing force, and tongue pressure-as independent variables, age-adjusted binomial logistic regression analysis was used to examine the effect of each oral function on the breathing patterns. Based on Akaike's Information Criterion (with Pin = Pout = 0.15)^54^, four independent variables were selected. Masticatory efficiency, which indicates the overall ability to perform various oral functions, was excluded. SPSS® version 25.0 (IBM, Chicago, IL, USA) was used for all statistical analysis, and statistical significance was set at 5%.

### Supplementary Information

Supplementary Table S1.

## Data Availability

The data that support the findings of this study are available from the corresponding author, upon reasonable request.

## References

[1] Feldman JL, Mitchell GS, Nattie EE (2003). Breathing: Rhythmicity, plasticity, chemosensitivity. Annu. Rev. Neurosci..

[2] McKay LC, Evans KC, Frackowiak RS, Corfield DR (2003). Neural correlates of voluntary breathing in humans. J. Appl. Physiol..

[3] Kral TRA (2023). Slower respiration rate is associated with higher self-reported well-being after wellness training. Sci. Rep..

[4] Lundberg JO, Weitzberg E, Lundberg JM, Alving K (1996). Nitric oxide in exhaled air. Eur. Respir. J..

[5] Kuroishi RC, Garcia RB, Valera FC, Anselmo-Lima WT, Fukuda MT (2015). Deficits in working memory, reading comprehension and arithmetic skills in children with mouth breathing syndrome: analytical cross-sectional study. Sao Paulo Med. J..

[6] Hunter SJ (2016). Effect of sleep-disordered breathing severity on cognitive performance measures in a large community cohort of young school-aged children. Am. J. Respir. Crit. Care Med..

[7] Bresolin D, Shapiro PA, Shapiro GG, Chapko MK, Dassel S (1983). Mouth breathing in allergic children: Its relationship to dentofacial development. Am. J. Orthod..

[8] Grippaudo C (2016). Association between oral habits, mouth breathing and malocclusion. Acta Otorhinolaryngol. Ital..

[9] Fraga WS, Seixas VM, Santos JC, Paranhos LR, César CP (2018). Mouth breathing in children and its impact in dental malocclusion: A systematic review of observational studies. Minerva Stomatol..

[10] Fujimoto S, Yamaguchi K, Gunjigake K (2009). Clinical estimation of mouth breathing. Am. J. Orthod. Dentofac. Orthop..

[11] Achmad H, Ansar AW (2021). Mouth breathing in pediatric population: A literature review. Annals Rom. Soc. Cell Biol..

[12] Sousa JB, Anselmo-Lima WT, Valera FC, Gallego AJ, Matsumoto MA (2005). Cephalometric assessment of the mandibular growth pattern in mouth-breathing children. Int. J. Pediatr. Otorhinolaryngol..

[13] Conti PB, Sakano E, Ribeiro MA, Schivinski CI, Ribeiro JD (2011). Assessment of the body posture of mouth-breathing children and adolescents. J. Pediatr..

[14] Nogami Y (2021). Prevalence of an incompetent lip seal during growth periods throughout Japan: A large-scale, survey-based, cross-sectional study. Environ. Health Prev. Med..

[15] Stupak, H. D. & Stupak, H. D. Strategies for addressing mouth-breathing treatment with an “adequate” nose. *Rethink. Rhinoplasty. Facial Surg*. 193–207 (2020).

[16] Takamoto K (2018). Lip closure training improves eating behaviors and prefrontal cortical hemodynamic activity and decreases daytime sleep in elderly persons. J. Bodyw. Mov. Ther..

[17] Tamura F (2020). Developmental problems concerning children's oral functions, based on a questionnaire administered to dentists and guardians. Pediatr. Dent. J..

[18] Ota C (2022). Predictors of developmental insufficiency of oral function in children. Pediatr. Dent. J..

[19] Japanese Association for Dental Science. Basic approach to developmental insufficiency of oral function. https://www.jads.jp/basic/pdf/document-200722-3.pdf. [Accessed 21 September 2023].

[20] Saitoh I (2017). The relationship between lip-closing strength and the related factors in a cross-sectional study. Pediatr. Dent. J..

[21] Basheer B, Hegde KS, Bhat SS, Umar D, Baroudi K (2014). Influence of mouth breathing on the dentofacial growth of children: A cephalometric study. J. Int. Oral Health..

[22] Sugawara M (1998). Effect of acute nasal obstruction on masticatory function. J. Masticat. Health Soc..

[23] Sakaguchi N, Ota I, Sugawara M, Asaka M, Igarashi S (1999). Effect of acute nasal obstruction on the masticatory function: With regard to masticatory efficiency. Jpn. J. Ped. Dent..

[24] Moss, M. L. The functional matrix. In: Vistas in orthodontics . (ed. Kraus, B. & Riedel, R.) 85–98 (Lea & Febiger, 1962).

[25] Humphreys HF, Leighton BC (1950). A survey of antero-posterior abnormalities of the jaws in children between the ages of 2 and 5 1/2 years of age. Br. Dent. J..

[26] Kogue Y, Igari K, Komatsu H, Mayanagi H (2003). Actual status mouth breathing in nursery school children. Jpn. J. Ped. Dent..

[27] Shindo Y (2009). On the connection between dentition/occlusion and mouth breathing in a primary school children: the analysis of a status survey in model primary school from a raising of children with normal occlusion project. Jpn. J. Ped. Dent..

[28] Souki BQ (2009). Prevalence of malocclusion among mouth breathing children: do expectations meet reality?. Int. J. Pediatr. Otorhinolaryngol..

[29] Zicari AM (2009). Oral breathing and dental malocclusions. Eur. J. Paediatr. Dent..

[30] Lessa FC (2005). Breathing mode influence in craniofacial development. Braz. J. Otorhinolaryngol..

[31] Emslie RD, Massler M, Zwemer JD (1952). Mouth breathing. I. Etiology and effects; a review. J. Am. Dent. Assoc..

[32] Sutcliffe P (1968). Chronic anterior gingivitis. An epidemiological study in school children. Br. Dent. J..

[33] Kawashima S (2000). Cephalometric comparisons of craniofacial and upper airway structures in young children with obstructive sleep apnea syndrome. Ear Nose Throat J..

[34] Boj, L., Catalá, M., García, C., Mendoza, A., Planells, P. & Odontopediatría. La Evolución del Niño al Adulto Joven, first ed., 522–523 (Ripano, 2010).

[35] Franco LP (2013). Is the growth pattern in mouth breathers comparable with the counterclockwise mandibular rotation of nasal breathers?. Am. J. Orthod. Dentofacial. Orthop..

[36] Chaves TC (2010). Craniocervical posture and hyoid bone position in children with mild and moderate asthma and mouth breathing. Int. J. Pediatr. Otorhinolaryngol..

[37] Chung LMI, Orta BP (2014). Comparison of cephalometric patterns in mouth breathing and nose breathing children. Int. J. Pediatr. Otorhinolaryngol..

[38] Mohamed AS (2022). Three-dimensional evaluation of hyoid bone position in nasal and mouth breathing subjects with skeletal Class I, and Class II. BMC Oral Health..

[39] Cuccia AM, Lotti M, Caradonna D (2008). Oral breathing and head posture. Angle Orthod..

[40] Behlfelt K, Linder-Aronson S, Neander P (1990). Posture of the head, the hyoid bone, and the tongue in children with and without enlarged tonsils. Eur. J. Orthod..

[41] Juliano ML, Machado MA, Carvalho LB, Prado LB, do Prado GF (2009). Mouth breathing children have cephalometric patterns similar to those of adult patients with obstructive sleep apnea syndrome. Arq. Neuropsiquiatr..

[42] Janicka A, Halczy-Kowalik L (2006). Hyoid bone position and tongue size and patency of upper airway structures. Ann. Acad. Med. Stetin..

[43] Ito D, Akashiba T, Yamamoto H, Kosaka N, Horie T (2001). Craniofacial abnormalities in Japanese patients with severe obstructive sleep apnoea syndrome. Respirology..

[44] Kikuchi M, Higurashi N, Miyazaki S, Itasaka Y (2000). Facial patterns of obstructive sleep apnea patients using Ricketts' method. Psychiatry. Clin. Neurosci..

[45] Saitoh I (2018). An exploratory study of the factors related to mouth breathing syndrome in primary school children. Arch. Oral Biol..

[46] Linder-Aronson, S. Effects of adenoidectomy on dentition and nasopharynx. *Trans. Eur. Orthod. Soc*. 177–186 (1972). 4523533

[47] Harvold EP, Tomer BS, Vargervik K, Chierici G (1981). Primate experiments on oral respiration. Am. J. Orthod..

[48] Miller AJ, Vargervik K, Chierici G (1984). Experimentally induced neuromuscular changes during and after nasal airway obstruction. Am. J. Orthod..

[49] Haishima H, Haishima K, Noda T (1997). Measurement of tongue and jaw movements during infant sucking. Jpn. J. Ped. Dent..

[50] Otani-Saito K, Ono T, Ishiwata Y, Kuroda T (2001). Modulation of the stretch reflex of jaw-closing muscles in different modes and phases of respiration. Angle Orthod..

[51] Inoue S, Tabuchi E, Imamura T, Noguchi M, Furuta I (2008). Influence of malalignment and malocclusion on mental and physical health-consciousness in senior high school students. Jpn. J. Public Health.

[52] McNamara JA (1984). A method of cephalometric evaluation. Am. J. Orthod..

[53] Iwasaki T (2022). Contribution to sleep medicine from dentistry: Sleep study of past and future. J. Oral Health. Biosci..

[54] Akaike, H. Information theory and an extension of the maximum likelihood principle. In Selected papers of hirotugu Akaike. 199–213 (Springer New York, 1998).

