# Supplementary material for: Mouth breathing reduces oral function in adolescence
Source: Sci Rep. 2024 Feb 15;14:3810. doi: 10.1038/s41598-024-54328-x (PMC10869721; doi:10.1038/s41598-024-54328-x)
Supplement: Supplementary file 1 — Supplementary Table S1. [file 41598_2024_54328_MOESM1_ESM.pdf]

Supplement File S1. Standardized cephalometric values in each breathing-pattern group

	Mouth-breathers	Oronasal-breathers	Nasal-breathers	P-value
SNA	-0.21 ± 0.99	0.01 ± 1.00	0.07 ± 1.01	0.229
SNB	-0.16 ± 1.13	-0.12 ± 0.91	0.11 ± 0.99	0.511
ANB	-0.09 ± 0.93	0.22 ± 0.95	-0.06 ± 1.05	0.812
FMA	0.46 ± 0.96	0.12 ± 0.96	-0.22 ± 0.98	0.014 *
ANS-Me	0.33 ± 0.97	0.26 ± 1.08	-0.24 ± 0.92	0.021 *
MP-H	0.14 ± 0.97	0.06 ± 1.04	-0.08 ± 1.00	0.387
C3-H	-0.37 ± 0.89	-0.05 ± 1.00	0.16 ± 1.02	0.023 *
Me-H	0.02 ± 0.94	-0.09 ± 1.11	0.03 ± 0.98	0.821
PNS-P	-0.38 ± 0.99	-0.16 ± 1.08	0.21 ± 0.93	0.021 *

* Comparison between mouth and nasal-breathers. Mann–Whitney U-test, $p<0.05$
